# Development of a Severity Score and Comparison With Validated Measures for Depression and Anxiety: Validation Study

**DOI:** 10.2196/30313

**Published:** 2021-11-10

**Authors:** William Lynch, Michael L Platt, Adam Pardes

**Affiliations:** 1 NeuroFlow, Inc Philadelphia, PA United States; 2 Department of Neuroscience Perelman School of Medicine University of Pennsylvania Philadelphia, PA United States; 3 Department of Psychology School of Arts and Sciences University of Pennsylvania Philadelphia, PA United States; 4 Marketing Department The Wharton School of Business University of Pennsylvania Philadelphia, PA United States

**Keywords:** PHQ-9, GAD-7, depression assessment, anxiety assessment, measurement-based care, integrated behavioral health

## Abstract

**Background:**

Less than 10% of the individuals seeking behavioral health care receive measurement-based care (MBC). Technology has the potential to implement MBC in a secure and efficient manner. To test this idea, a mobile health (mHealth) platform was developed with the goal of making MBC easier to deliver by clinicians and more accessible to patients within integrated behavioral health care. Data from over 3000 users of the mHealth platform were used to develop an output severity score, a robust screening measure for depression and anxiety.

**Objective:**

The aim of this study is to compare severity scores with scores from validated assessments for depression and anxiety and scores from clinician review to evaluate the potential added value of this new measure.

**Methods:**

The severity score uses patient-reported and passively collected data related to behavioral health on an mHealth platform. An artificial intelligence–derived algorithm was developed that condenses behavioral health data into a single, quantifiable measure for longitudinal tracking of an individual’s depression and anxiety symptoms. Linear regression and Bland-Altman analyses were used to evaluate the relationships and differences between severity scores and Personal Health Questionnaire-9 (PHQ-9) or Generalized Anxiety Disorder-7 (GAD-7) scores from over 35,000 mHealth platform users. The severity score was also compared with a review by a panel of expert clinicians for a subset of 250 individuals.

**Results:**

Linear regression results showed a strong correlation between the severity score and PHQ-9 (*r*=0.74; *P*<.001) and GAD-7 (*r*=0.80; *P*<.001) changes. A strong positive correlation was also found between the severity score and expert panel clinical review (*r*=0.80-0.84; *P*<.001). However, Bland-Altman analysis and the evaluation of outliers on regression analysis showed that the severity score was significantly different from the PHQ-9.

**Conclusions:**

Clinicians can reliably use the mHealth severity score as a proxy measure for screening and monitoring behavioral health symptoms longitudinally. The severity score may identify at-risk individuals who are not identified by the PHQ-9. Further research is warranted to evaluate the sensitivity and specificity of the severity score.

## Introduction

### Integrated Measurement-Based Behavioral Health Care

Measurement-based care (MBC) can be broadly defined as the use of continuous monitoring of patient data to inform and, as needed, redirect clinical care [1]. Behavioral health focuses on how behaviors impact a person’s health both physically and mentally. Within the behavioral health field, MBC is considered an evidence-based practice, which is defined as relying on the use of data from controlled scientific studies reported in the published literature to make reasonable and conscientious decisions about clinical care [2]. As a framework for behavioral health care, MBC uses validated clinical assessments to measure symptoms of anxiety and depression before or during a patient-clinician interaction, and clinicians use these measurements to guide further clinical interventions [1]. MBC often occurs during cognitive behavioral therapy (CBT) or other psychological treatments [1,2]. Despite strong evidence that MBC improves behavioral health outcomes [1,3-6], less than 20% of behavioral health care providers use MBC [5,6]. Research has shown that barriers to the use of MBC include, but are not limited to, the costs in both time and resources to take measurements in addition to concerns about potential violations of confidentiality with paper and pen measurements [4,5].

Integrating behavioral health care into primary care has been proposed as a means of improving access to behavioral health care and is supported by both the American Association of Family Physicians and the American Psychiatric Association [7-10]. Globally, the integrated behavioral health model has demonstrated success in the United Kingdom with the web-based Beating the Blues program [11]. The program uses CBT and has been shown to be evidence-based and cost-effective in treating anxiety and depression by the primary care provider [11]. In the integrated behavioral health model, the primary care clinician screens patients for symptoms of mental health disorders at every visit and recommends and prescribes further assessment and treatment as needed, whether that be with medication, CBT, or other psychological treatments [9,10]. Patients are followed up by a care manager, and psychiatrists are available to consult with the primary care clinician as needed for any case. The integrated behavioral health model could help address the shortage of behavioral health care providers and low levels of access to behavioral health care, as approximately 75% of people in the United States have a primary care provider [12]. Globally, this percentage rises to 85% of people with primary care providers [13], demonstrating the imperative need to use primary care providers for behavioral health. Integrated behavioral health may also help address the unequal distribution of behavioral health care providers, as this model allows for remote screening and consultations. However, the integrated behavioral health model is not yet widely used, as only 4.3% of the primary care visits in 2016 included screening for behavioral health symptoms [14].

Given such low levels of depression or anxiety screening during primary care visits, it is perhaps not surprising that data are limited regarding the use of MBC within integrated behavioral health care. However, it is known that time, resources, and confidentiality are obstacles to the use of MBC by behavioral health providers who typically have 45 minutes with a patient weekly or biweekly [4,5]. There is also a paucity of data regarding the amount of time primary care physicians spend with individual patients. Typically, visits are scheduled every 15 minutes, and data suggest that the average visit may last 17-21 minutes [15,16], although a time-and-motion study suggests that only 53% of that time (9-11 minutes) is spent engaging with the patient directly [17]. Although current validated measures of depression and anxiety—the Personal Health Questionnaire-9 (PHQ-9) and the Generalized Anxiety Disorders-7 (GAD-7)—are brief and take ≤5 minutes to administer, providing MBC might take away 20% to 60% of the limited face-to-face time, during which many other clinical tasks must also be performed.

### Unmet Behavioral Health Care Needs

Whether because of access to care, short time spent with physicians, or shortage of behavioral health clinicians, many individuals with depression and anxiety do not receive MBC, despite evidence that it improves outcomes [1]. Moreover, considering the epidemic proportions of depression and anxiety and related disability and mortality, there is an urgent need to find novel ways for primary care clinicians to provide comprehensive MBC in an integrated behavioral health care model. In addition, although the PHQ-9 and GAD-7 function efficiently on their own to identify at-risk patients, they alone may not capture the full scope of potential indicators of an at-risk patient beyond questions asked in the screening assessment.

Therefore, a mobile health (mHealth) platform was built with the goal of improving access to MBC at scale in an integrated behavioral health care model. There are some common concerns regarding smartphone apps for behavioral health care. For example, digital measurements are not necessarily evidence-based, may not provide treatment that is equivalent to that received from a trained clinician, and often have a low frequency of use and engagement by patients. Often, the measures used in apps were originally validated as paper and pen or pencil instruments, and it is not clear if digital versions have the same sensitivity and specificity. The number of available behavioral health apps for smartphones is in the tens of thousands [18], whereas a search on PubMed for clinical studies of such apps returns results in the thousands. The apps also deliver a wide variety of measures and interventions, making comparisons among apps and between apps and human-delivered (in-person or via telehealth) clinical interventions difficult, if not impossible, without such systematic studies. Concerns about the persistence of any effects are related to the fact that the use of behavioral health apps, on average, falls off precipitously, with only 3.3% of downloaded behavioral health apps used for more than 30 days [19]. As part of an effort to continue simplifying and increasing the adoption of MBC by clinicians and patients, a severity score was developed within the abovementioned mHealth platform. The severity score is a single composite, relative nondiagnostic measure of behavioral health that can be tracked over time.

To understand the role of the severity score in MBC, the aim of this study is to validate the output measure against established scores of behavioral health assessments. Herein, we report the data comparing the severity score with scores from the PHQ-9 assessment, the GAD-7 assessment, and expert clinician consensus.

## Methods

### The mHealth Platform

In this study, all measurements were taken on the NeuroFlow mHealth platform (NeuroFlow, Inc), where all measurements were completed by patients on their own devices. Through the NeuroFlow app or desktop interface, patients record, track, and report their mood, sleep quality, stress level, and complete PHQ-9 and GAD-7 assessments regularly. The app also provides engaging educational videos based on users’ self-reported scores and symptom changes over time. Clinicians with access to the mHealth platform send individualized links to their patients, which allows the patient to download the mobile app on their smartphone or access the platform through a website.

Although the latter features are not necessarily interventional or measurement oriented, they serve the goal of maintaining user engagement and lasting behavior change over time by supporting users. All measurements were securely stored in the Health Insurance Portability and Accountability Act (HIPAA)-compliant database, and when in use, a clinician accessed only their own patients’ information via a digital dashboard that integrates with the electronic medical record. This integration ensured that taking the measurements required for MBC does not further reduce the already small amount of time that clinicians have with patients. Instead of performing screening assessments, they can immediately see which of their patients may require a behavioral health intervention. The reporting system in the mHealth platform alerts clinicians about the patients who are not progressing or who have declining behavioral health. The features of the mobile app include daily self-rating scales for stress, mood, sleep, and pain; mindfulness tools; general health education; and step tracking. Among all mHealth app use, 30-day user retention is 70% compared with a typical 30-day retention rate of 3.3% for behavioral health mobile apps [20].

### Development of the Severity Score

**Figure 1:**
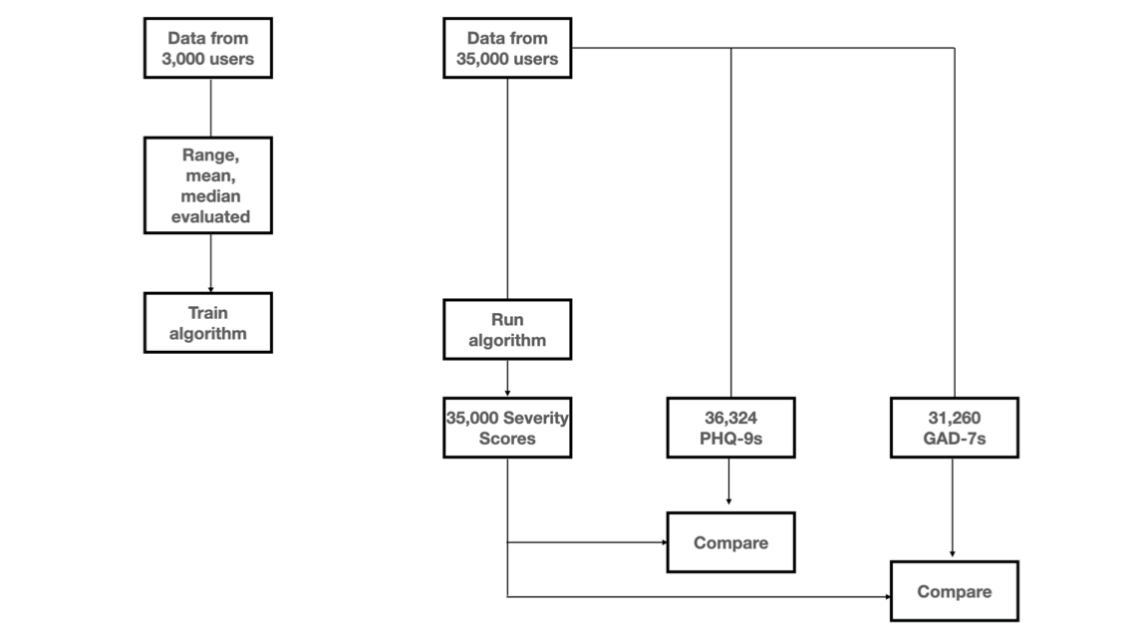
Overview of the process for developing and assessing the severity score. GAD-7: Generalized Anxiety Disorder-7; PHQ-9: Personal Health Questionnaire-9.

To create the composite severity score, we developed a proprietary algorithm that uses the measures recorded in the mHealth platform NeuroFlow by patients. The data set included deidentified records of over 3000 individuals who used the mHealth platform after it was assigned to them by a clinician between 2018 and 2019. Weighted variables included total scores on the PHQ-9 (27-point scale) or GAD-7 (21-point scale) measures taken within the app, self-reported sleep quality measures (scale of 0-10), self-reported mood measures (scale of 0-10), active behavioral health treatment (yes or no), frequency of an individual using specific activities within the app (collected passively), and whether the individual had endorsed having suicidal ideation (yes or no). For each measure of the severity score, descriptive statistics were calculated to determine the distribution of variables; central tendencies were calculated through mean and median and the spread of variable values through range, SD, and variance. Variables were assigned positive or negative weights based on expert clinician input, and these variables were combined to produce a severity score measure, using a proprietary artificial intelligence algorithm. Severity score measures ranged from 1 to 5, with 1 indicating a low risk for common behavioral health conditions (eg, depression and anxiety) and 5 indicating a high risk for such conditions (Figure 1).

### Comparisons With Validated Assessments

The PHQ-9 is a 9-item questionnaire used to screen for depression in medical settings. Each individual item is scored as a potential total score from 0 to 27. PHQ-9 scores of 5, 10, 15, and 20 represent mild, moderate, moderately severe, and severe depression, respectively. Under the scope of MBC, patients are asked by their care provider to complete assessments every 2 to 4 weeks. The clinical purpose of these assessments is to help support clinicians in making a diagnosis, to quantify depression symptoms, and to monitor changes over time to determine if treatment is making a difference [21,22]. The GAD-7 is a 7-item questionnaire used to screen for anxiety in medical settings; individuals are asked how often they have experienced certain feelings in the last 2 weeks on a scale of 0- 3. The total possible score is 21, and scores of 5, 10, and 15 are considered cut-offs for the presence of mild, moderate, and severe anxiety, respectively [23]. These are components of the measurements that patients complete in the mHealth platform such that for every severity score logged in the platform, there is a corresponding overall PHQ-9 or overall GAD-7 score for that individual at that point in time.

Using deidentified records, the severity score for a given individual was plotted against their GAD-7 (n=31,260) and PHQ-9 (n=36,324) scores. For each comparison, we used linear regression to fit the slope of the line to our data and used Pearson product-moment correlation coefficient (r) to quantify and summarize the direction and magnitude of the relationship between our variables. The Pearson product-moment correlation coefficient r can be any number between −1 and 1. The sign of r corresponds to the direction of the relationship between the variables and the number corresponding to the magnitude of the relationship between the variables.

The severity score measure and PHQ-9 scores were also compared using the Bland-Altman analysis, which compares the differences between 2 measures versus the mean of the 2 measures [24]. This analysis evaluated whether the two measurement methods returned the same results and therefore could be used interchangeably [25]. Because PHQ-9 scores vary from 0 to 27 and severity score measures vary from 1 to 5, we first converted PHQ-9 scores to the clinically meaningful categories of 1 for no depression (scores 0-4 on PHQ-9), 2 for mild depression (scores 5-9 on PHQ-9), 3 for moderate depression (scores 10-14 on PHQ-9), 4 for moderately severe depression (scores 15-19 on PHQ-9), and 5 for severe depression (scores 20-27 on PHQ-9). The differences between the converted PHQ-9 score and the severity score were then plotted against the average of the converted PHQ-9 and the severity score. To create the change scores for the severity score, we took the absolute change between a severity score taken at time point *t* and the previous severity score taken at time point *t-1* for a given participant. We then applied the same calculation to create the change scores for the PHQ-9 and the GAD-7.

### Comparison With Clinical Reviews

A panel of 6 behavioral-health expert clinicians, including licensed psychologists, licensed clinical social workers, and 1 mental health psychiatric board–certified registered nurse, provided 2 clinical reviews for each of the 250 individuals based on 2 different blinded presentations of data from deidentified patient records. As shown in Table 1, the first data set provided only 30-day average PHQ-9 and GAD-7 scores recorded in the app over a 30-day period for these 250 individuals. The second data set provided measures included in the severity score in addition to the 30-day average and maximal PHQ-9 and GAD-7 scores, including the suicidal ideation score from the PHQ-9, measures of sleep and mood, whether the individual reported working with a behavioral health specialist, and whether severe depression or anxiety had been present in the last 30-day period. The 2 data sets were randomized independently to ensure that the order of the records was not repeated. Clinicians were asked to assign a clinical rating of symptom severity to each of the 250 individual records for both data sets using a scale of 1 (low to minimal) to 5 (severe) (Textbox 1).

**Table 1:** Variables included in data set 2 evaluated by expert panel.

Variable	Possible scores^a^	Description
avg_phq9_score	0-27	The average PHQ-9^b^ score
max_phq9_score	0-27	The highest PHQ-9 score recorded
q9_max	0-3	The highest score recorded for the ninth question of the PHQ-9, which screens for suicidal ideation as follows: “Over the past 2 weeks, how often have you been bothered by thoughts that you would be better off dead or of hurting yourself in some way?” (0=not at all, 1=several days, 2=more than half the days, and 3=nearly every day)
avg_gad7_score	0-21	The average GAD-7^c^ score
max_gad7_score	0-21	The highest GAD-7 score recorded
avg_sleep_rating	0 (best)-4 (worst)	The average sleep rating
sleep_count	0-30	The number of sleep ratings
avg_mood_rating	0 (best)-4 (worst)	The average mood rating
mood_count	0-30	The number of mood ratings
has_bh_specialist	0=no and 1=yes	This value denotes whether a patient self-reported having a behavioral health clinician
is_severe^d^	0=no and 1=yes	This value denotes whether a severe GAD-7 or PHQ-9 score was recorded in the 30-day period before this period

^a^All scores except *is_severe* are for the same 30-day period.

^b^PHQ-9: Physical Health Questionnaire-9.

^c^GAD-7: General Anxiety Disorders-7.

^d^*is_severe* is gathered from the last 30-day period.

**Clinician symptom severity rating scale.**

**Scores**

1: low to minimal severity

2: mild severity

3: moderate severity

4: moderately severe

5: severe

**Figure 2:**
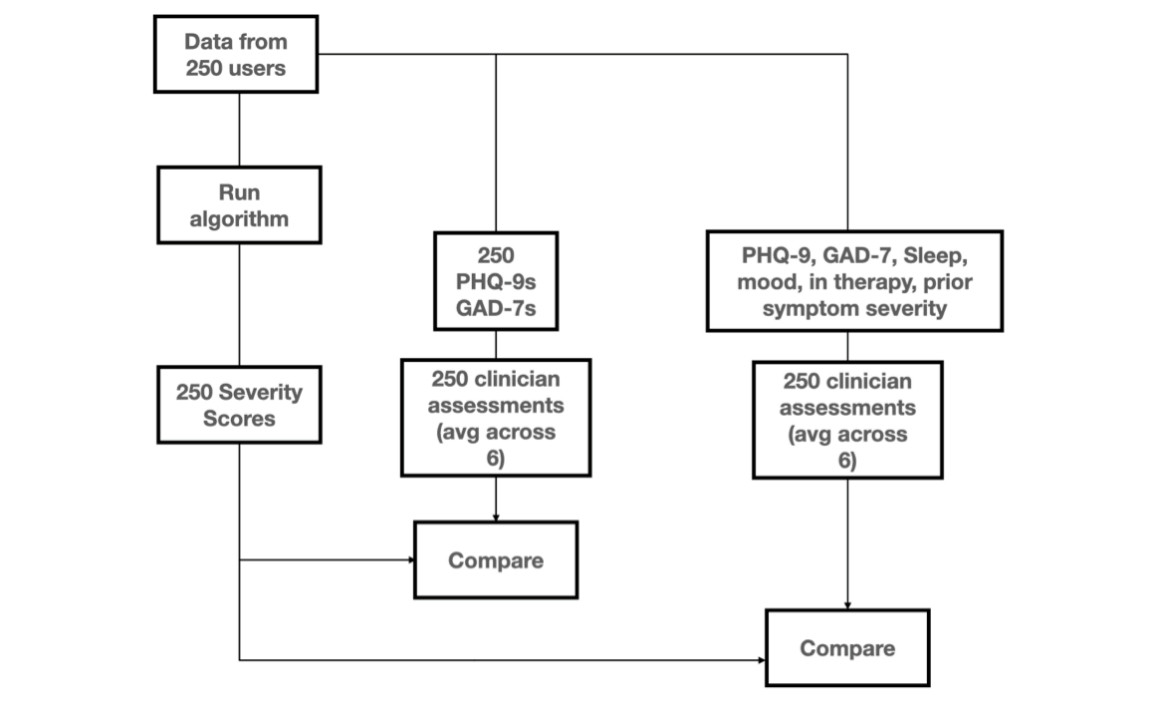
Overview of the process for comparing the severity score with clinicians’ reviews. avg: average; GAD-7: Generalized Anxiety Disorder-7; PHQ-9: Personal Health Questionnaire-9.

For each data set, the mean clinician expert score was plotted against the severity score for the same individual (n=250). Linear regression was calculated, and both the Pearson correlation coefficient and the Kendall tau measure were used to evaluate the relationship between the mean clinician expert score and the severity score (Multimedia Appendix 1). Figure 2 provides an overview of how the severity score was compared with clinical reviews.

## Results

### Comparison of the Severity Score With Validated Assessments

**Figure 3:**
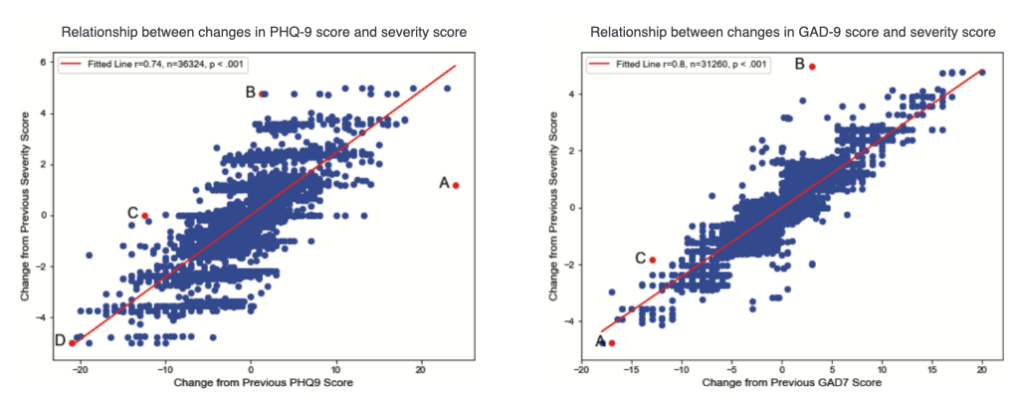
Change in severity score versus change in Personal Health Questionnaire-9 (PHQ-9) score or Generalized Anxiety Disorders-7 (GAD-7) over a 30-day period.

Normal distribution of data points around the mean and median were found for all 3 changes measured (ie, severity score, PHQ-9, and GAD-7). This study reviewed the changes in scores to better understand the relationship between severity scores and standardized assessments. The data for each measure were homoscedastic, supporting the use of linear regression for the analysis of possible correlations. After plotting the change in severity score versus the change in PHQ-9 scores for each individual record, we found a strong positive correlation between change in severity score and change in PHQ-9 score (*r*=0.74; *P*<.001; Figure 3). Similarly, changes in the severity score were strongly and positively correlated with changes in the GAD-7 score (*r*=0.80; *P*<.001; Figure 3).

We also evaluated several data points that did not fit the linear regression and found that these were not errors or anomalies but rather were cases in which the severity score provided clinically meaningful information not captured by the PHQ-9 or GAD-7 score alone. For example, an individual whose PHQ-9 score changed from indicating minimal to moderately severe symptoms of depression (Figure 3) had a smaller change in the severity score because the severity score measure incorporated their active involvement in mental health treatment and absence of suicidal ideation. In contrast, another individual had PHQ-9 score changes that reflected a decrease in symptoms of depression but did not have a drop in the severity score because the severity score incorporated ongoing thoughts of self-harm or suicidal ideation reported by this individual.

**Figure 4:**
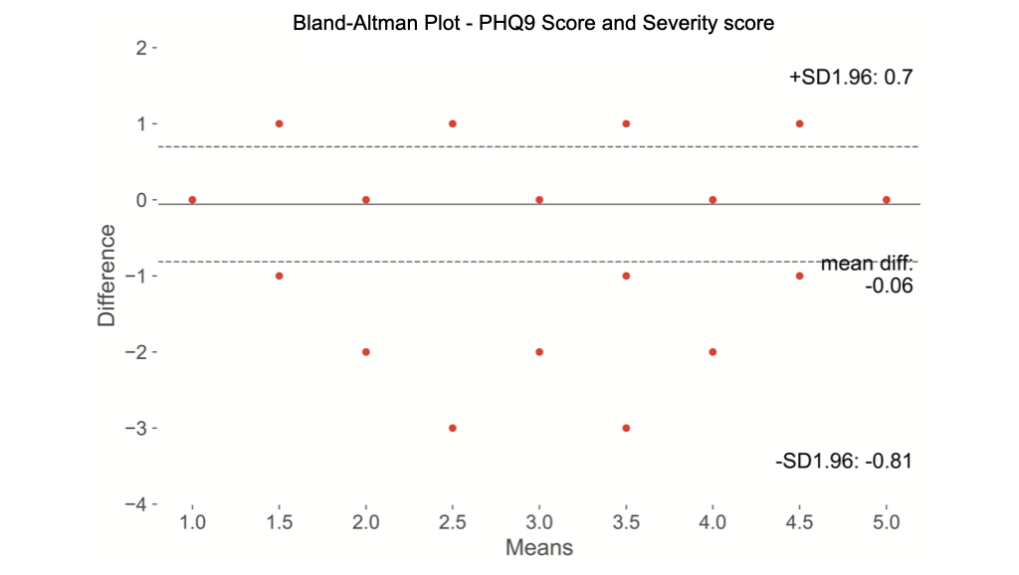
Bland-Altman analysis shows that the Personal Health Questionnaire-9 (PHQ-9) and the severity score are significantly different measures with differences increasing around the mean of the 2 measures.

On the Bland-Altman analysis, comparing the PHQ-9 with the severity score, we found that the severity score measure was significantly different from the PHQ-9, with the difference between the two measures increasing around the mean of the two measures (Figure 4).

### Comparison With Clinician Review

**Figure 5:**
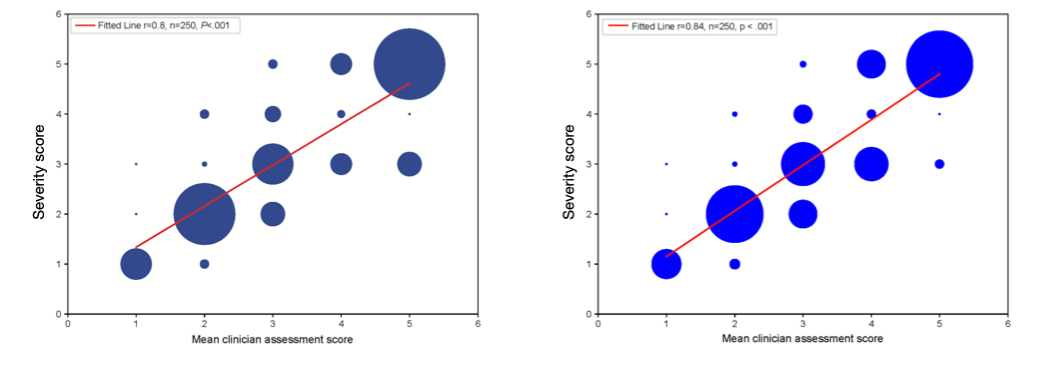
Severity score compared with mean clinical review score by expert clinician panel.

There were also strong correlations between the severity score and clinical experts’ reviews both when the expert clinical score was generated from only average PHQ-9 and GAD-7 scores (*r*=0.80; *P*<.001; Figure 5) and when the larger data set was used by the clinicians (*r*=0.84; *P*<.001; Figure 5). Linear regression analysis showed a strong correlation between the severity score and clinician reviews for both data sets, with a slightly stronger correlation for the more comprehensive data set (*r*=0.84; *P*<.001) versus that for just the average PHQ-9 and GAD-7 scores (*r*=.80, *P*<.001). The 2 data sets came from the same 250 individuals and were separately randomized to ensure that the records were presented in different orders.

## Discussion

### Principal Findings

This study examined a new measure to be used in comprehensive MBC screening that considers validated assessments, subjective self-assessments, ongoing treatment, and app activity to gain full insight into the lives of patients. This study found relationships among the severity score, PHQ-9, and GAD-7. There was also a strong positive correlation between the severity score and expert panel clinical review, indicating that the severity score may serve as a relative measure of depression or anxiety. Findings from this study can inform further development of a comprehensive measure for clinicians to further identify at-risk individuals who may have otherwise been overlooked or not treated.

Integrating MBC into primary care has proven to be effective, but it is still not widely adopted [7]. For example, simply screening for the presence of depression or anxiety was performed in only 4.3% of primary care visits in 2016 [13]. Barriers to the implementation of MBC in primary care include systemic factors such as the lack of a consistently used measurement system and limited resources for training staff to use a measurement system in a HIPAA-compliant manner. In behavioral health care, HIPAA compliance is of particular importance because of the stigma surrounding behavioral health disorders that leads to discrimination and decreased quality of life [3-6,8,9,25]. At the individual patient level, concerns about confidentiality and the time burden for completing measurements are major barriers to the use of MBC. At the provider level, it is difficult to find time to measure behavioral health symptoms during primary care visits, given the high number of clinical tasks that must be prioritized and completed. Measurement-based care platforms and rating measures such as the severity score were developed with the goal of facilitating MBC integration into primary care by reducing time burden. For example, these types of platforms and rating scales take the measurement aspect outside of the clinical appointment and into the patients’ daily activities with an engaging smartphone or desktop app.

Before investigating the feasibility and usability of the severity score in a clinical setting, it was crucial to first understand whether the severity score added value beyond the information provided by the PHQ-9 and the GAD-7, resulting in a more comprehensive understanding of the behavioral health profile of the user. The severity score is derived using an artificial intelligence–powered algorithm that uses both patient-reported measures taken outside the clinical visit and passively collected data through the use of an app. Regression analysis showed that the severity score correlated strongly with both the PHQ-9 and the GAD-7 scores with 0.88 sensitivity and specificity and 0.83 sensitivity and 0.84 specificity, respectively [21-23]. The severity score correlated strongly with clinician review based on PHQ-9 and GAD-7 scores alone (*r*=0.80; *P*<.001) and based on the analysis of a larger number of data reported using the mHealth platform (*r*=0.84; *P*<.001). These strong correlations suggest that the severity score can be used as a proxy measure for the presence of symptoms of depression and anxiety.

For example, if a clinician observed (on the clinician dashboard or electronic health record) that a patient had a severity score of 2 or more, it would prompt them to address depression or anxiety and perhaps schedule a separate follow-up visit for these concerns, as needed. Although the expert clinician panel’s assessments in this study were made by reviewing recorded data measures rather than in-person clinical assessments, the strong correlations with both clinician review and validated measures shows promise in the severity score for clinical use.

Findings from the Bland-Altman analysis revealed that the severity score is significantly different from the PHQ-9, which likely reflects the larger number of measures and the use of weighting for more or less significance for depression or anxiety based on comparison with a large set of individuals. Analysis of outliers in the regression analysis showed instances in which the severity score more accurately identified people with or without clinically significant depression or anxiety than the PHQ-9 or GAD-7 scores, respectively. The identification of the severity score as a measure that is different but still correlates with both validated assessments and clinician review suggests that it can be used as a proxy for those validated measures and that it may have greater utility than those measures alone.

Although combined items from the PHQ-9 and GAD-7 comprise only 16 questions, administering these measures during the primary care visit can be challenging because of time constraints. For example, the addition of just one of these 2 measures could take 20%-40% of the available time of an already short primary care visit lasting 17-21 minutes. Rather than shortening the time needed for measurement, mHealth platforms such as NeuroFlow can aid in MBC integration by taking time for measurement out of the clinical visit. Using the data reported by patients through the use of the app and passively collected data, the severity score provides a measure that is inclusive not only of the full PHQ-9 and GAD-7 data but also other meaningful measures. By assessing a larger number of parameters with an artificial intelligence–driven algorithm, the severity score may be able to identify at-risk individuals who may have been missed with only a PHQ-9 or GAD-7 assessment. The reason for this is the severity score number factoring in multiple assessments and variables, including subjective variables such as mood or sleep. For example, if an individual is not sleeping well, this might be evident in the severity score, whereas it may not be in the PHQ-9 or GAD-7 scores. In addition, digital delivery of the severity score in the patient’s electronic health record confers advantages such as HIPAA-compliant screening, mitigation of confidentiality concerns, and completed assessments before meeting with the clinician. This may allow clinicians to use additional time for further assessment and suggestions for treatment, as appropriate.

Providing a system-wide, HIPAA-compliant measurement system that requires minimal training of medical practice personnel can be delivered in a digital manner, and integrating measurement-based care (MBC) into primary care practices confers several advantages. From the clinicians’ perspective, the severity score is valuable because it provides a single measure ranging from 1 to 5 that is generated from daily platform use by the patient, can be integrated into the electronic health record, and can alert the clinician to the presence of concerning endorsements of depression and anxiety symptoms, which in turn can prompt further assessment, treatment, and referrals, as needed. Another advantage of the severity score is that it seamlessly incorporates the measurement of behavioral health symptoms into a person’s typical day-to-day activities that include the use of their mobile phones. Taking some time (approximately 30 seconds) to track mood or sleep for multiple days of the week is less onerous for the patient than taking 4 to 8 minutes needed for both the PHQ-9 and GAD-7.

### Strengths and Limitations

As with all health-related apps, there is appropriate concern regarding whether individuals will use the app and record data consistently. In this regard, the severity score demonstrated 2 advantages. First, the platform has a high user retention rate of 70%, which is 21 times higher than the typical 30-day retention rate of 3.3% for health-related apps [20]. The platform has user retention rates of 32% and 27% at 6 and 12 months, respectively, and receives a Net Promoter Score of 41, which is 14 points higher than the industry average [26]. This is achieved through well-tested patient engagement techniques that leverage an omni-channel communication strategy. The platform sends patient notifications to register and reminders to engage with the assigned content and activities. Notifications can be in the form of email, SMS text messages, and push notifications depending on the user’s preferences. Second, the incorporation of additional patient-reported measures (eg, sleep rating, mood rating, and number of times a measure was tracked) in addition to PHQ-9 and GAD-7 scores means that a reliable severity measure is available even if 1 measure (eg, the PHQ-9 or GAD-7 score) is not present for a particular individual.

As with all studies, this study is also not without its limitations. For example, given the retrospective design, the sample is not always representative of the population as a whole, and the data set may be at risk of recall bias. Future research with a much larger sample size may prove fruitful, in addition to a prospective study design.

### Conclusions

The severity score is a screening measure of the symptoms of depression and anxiety and other important variables, such as mood and sleep, generated with an artificial intelligence–powered algorithm developed from the analysis of 3000 mHealth users. A comparison of over 35,000 severity scores with PHQ-9 and GAD-7 scores taken at the same point in time by the same individuals demonstrated a strong correlation between the severity score and scores from both the PHQ-9 and GAD-7. Clinician reviews of the PHQ-9 and GAD-7 scores also correlate strongly with the severity score. Together, these correlations strongly suggest that the severity score can be used as a proxy measure for the presence of depression and anxiety. However, the Bland-Altman analysis shows that the severity score is a significantly different output measure. Prospective feasibility studies to further measure the sensitivity, specificity, and clinical noninferiority of the severity score are warranted.

## Appendix

Multimedia Appendix 1 Multiple linear regression validation.

## Abbreviations

GAD-7: Generalized Anxiety Disorder-7
HIPAA: Health Insurance Portability and Accountability Act
MBC: measurement-based care
mHealth: mobile health
PHQ-9: Personal Health Questionnaire-9
