# Supplementary figures and images for: Development of a Severity Score and Comparison With Validated Measures for Depression and Anxiety: Validation Study
Source: JMIR Form Res. 2021 Nov 10;5(11):e30313. doi: 10.2196/30313 (PMC8663615; doi:10.2196/30313)

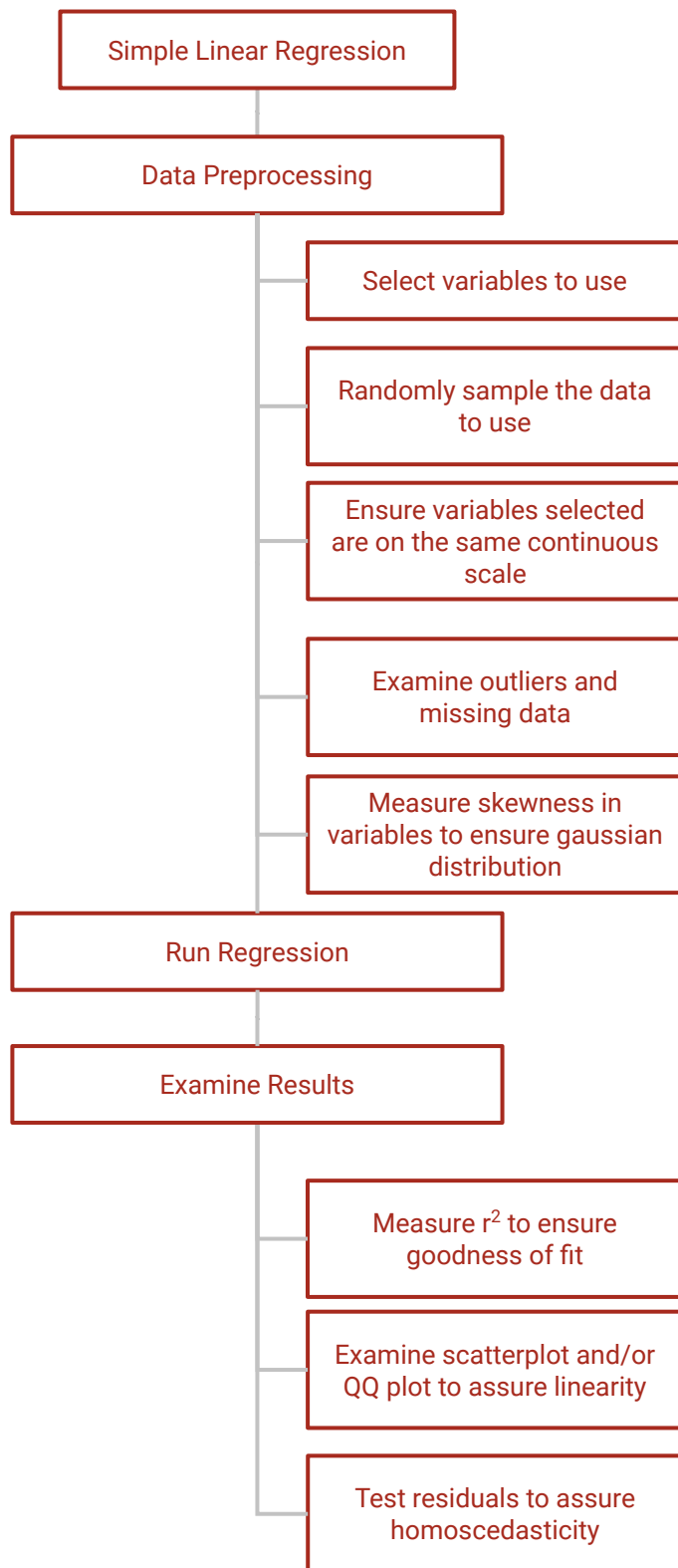

Supplement: Multimedia Appendix 1 [file formative_v5i11e30313_app1.pdf]
